# Supplementary material for: HIVprotI: an integrated web based platform for prediction and design of HIV proteins inhibitors
Source: J Cheminform. 2018 Mar 9;10:12. doi: 10.1186/s13321-018-0266-y (PMC5845081; doi:10.1186/s13321-018-0266-y)
Supplement: Supplementary file 2 — Additional file 2. Source code of HIVProtI web server. [file 13321_2018_266_MOESM2_ESM.zip › HIVprotI_Source-code/hivprotI/weka-3-6-6/doc/weka/core/Drawable.html]

Drawable


---


|  |  |  |
| --- | --- | --- |
| **Overview** \| **Package** \| **Class** \| **Tree** \| **Deprecated** \| **Index** \| **Help** \| **Weka's home** | |  |
| **PREV CLASS**   **NEXT CLASS** | **FRAMES**    **NO FRAMES**     **All Classes** |  |
| SUMMARY: NESTED \| FIELD \| CONSTR \| METHOD | DETAIL: FIELD \| CONSTR \| METHOD |  |


---


## weka.core Interface Drawable

**All Known Implementing Classes:**: ADTree, AttributeSelectedClassifier, BayesNet, BayesNetGenerator, BIFReader, C45PruneableClassifierTree, C45PruneableClassifierTreeG, ClassifierTree, Cobweb, CostSensitiveClassifier, CVParameterSelection, EditableBayesNet, FilteredClassifier, FT, HierarchicalClusterer, J48, J48graft, LADTree, LMT, M5P, NBTree, NBTreeClassifierTree, PruneableClassifierTree, RandomTree, REPTree, ThresholdSelector, UserClassifier

---

``` public interface Drawable ```

Interface to something that can be drawn as a graph.

**Version:**
:   $Revision: 6589 $

**Author:**
:   Ashraf M. Kibriya(amk14@cs.waikato.ac.nz), Eibe Frank(eibe@cs.waikato.ac.nz)

---

| **Field Summary** | |
| --- | --- |
| `static int` | `BayesNet` |
| `static int` | `Newick` |
| `static int` | `NOT_DRAWABLE` |
| `static int` | `TREE` |


| **Method Summary** | |
| --- | --- |
| `java.lang.String` | `graph()`             Returns a string that describes a graph representing the object. |
| `int` | `graphType()`             Returns the type of graph representing the object. |

**Field Detail**

### NOT\_DRAWABLE

```
static final int NOT_DRAWABLE
```

**See Also:**: Constant Field Values

---


### TREE

```
static final int TREE
```

**See Also:**: Constant Field Values

---


### BayesNet

```
static final int BayesNet
```

**See Also:**: Constant Field Values

---


### Newick

```
static final int Newick
```

**See Also:**: Constant Field Values


**Method Detail**

### graphType

```
int graphType()
```

:   Returns the type of graph representing
    the object.

    :   **Returns:**: the type of graph representing the object

---


### graph

```
java.lang.String graph()
                       throws java.lang.Exception
```

:   Returns a string that describes a graph representing
    the object. The string should be in XMLBIF ver.
    0.3 format if the graph is a BayesNet, otherwise
    it should be in dotty format.

    :   **Returns:**: the graph described by a string **Throws:**: `java.lang.Exception` - if the graph can't be computed


---


|  |  |  |
| --- | --- | --- |
| **Overview** \| **Package** \| **Class** \| **Tree** \| **Deprecated** \| **Index** \| **Help** \| **Weka's home** | |  |
| **PREV CLASS**   **NEXT CLASS** | **FRAMES**    **NO FRAMES**     **All Classes** |  |
| SUMMARY: NESTED \| FIELD \| CONSTR \| METHOD | DETAIL: FIELD \| CONSTR \| METHOD |  |


---
